# Involvement of Complexin 2 in Docking, Locking and Unlocking of Different SNARE Complexes during Sperm Capacitation and Induced Acrosomal Exocytosis

**DOI:** 10.1371/journal.pone.0032603

**Published:** 2012-03-06

**Authors:** Pei-Shiue J. Tsai, Ian A. Brewis, Jillis van Maaren, Bart M. Gadella

**Affiliations:** 1 Department of Farm Animal Health, Graduate School of Animal Health, Faculty of Veterinary Medicine, Utrecht University, Utrecht, the Netherlands; 2 Department of Biochemistry and Cell Biology, Graduate School of Animal Health, Faculty of Veterinary Medicine, Utrecht University, Utrecht, the Netherlands; 3 Cardiff University School of Medicine, Cardiff, United Kingdom; Institute of Molecular and Cell Biology, Singapore

## Abstract

Acrosomal exocytosis (AE) is an intracellular multipoint fusion reaction of the sperm plasma membrane (PM) with the outer acrosomal membrane (OAM). This unique exocytotic event enables the penetration of the sperm through the zona pellucida of the oocyte. We previously observed a stable docking of OAM to the PM brought about by the formation of the *trans*-SNARE complex (syntaxin 1B, SNAP 23 and VAMP 3). By using electron microscopy, immunochemistry and immunofluorescence techniques in combination with functional studies and proteomic approaches, we here demonstrate that calcium ionophore-induced AE results in the formation of unilamellar hybrid membrane vesicles containing a mixture of components originating from the two fused membranes. These mixed vesicles (MV) do not contain the earlier reported trimeric SNARE complex but instead possess a novel trimeric SNARE complex that contained syntaxin 3, SNAP 23 and VAMP 2, with an additional SNARE interacting protein, complexin 2. Our data indicate that the earlier reported raft and capacitation-dependent docking phenomenon between the PM and OAM allows a specific rearrangement of molecules between the two docked membranes and is involved in (1) recruiting SNAREs and complexin 2 in the newly formed lipid-ordered microdomains, (2) the assembly of a fusion-driving SNARE complex which executes Ca^2+^-dependent AE, (3) the disassembly of the earlier reported docking SNARE complex, (4) the recruitment of secondary zona binding proteins at the zona interacting sperm surface. The possibility to study separate and dynamic interactions between SNARE proteins, complexin and Ca^2+^ which are all involved in AE make sperm an ideal model for studying exocytosis.

## Introduction

Capacitation and acrosomal exocytosis (AE, also known as the acrosome reaction, AR) are processes required for sperm to fertilize the oocyte *in vivo*. A number of changes occur at the sperm surface during sperm capacitation [1]. These capacitation-induced surface changes are effectuated *in vitro* by two capacitation factors, namely bicarbonate and fatty acid free bovine serum albumin (BSA). In the presence of calcium, both factors activate protein kinase A (PKA) and tyrosine kinase signaling pathways that lead to the hypermotility of sperm cells and the specific depletion of cholesterol from the sperm surface [2]–[4]. The depletion of cholesterol from the sperm plasma membrane (PM) allows the rearrangement of proteins and lipids and leads to an increase in membrane fluidity. Furthermore, a change in the influx and efflux of essential ions (e.g. calcium, potassium) through the activated ion channels alter the membrane potential [5], [6] and this activates relevant processes that are required for the execution of AE [7], [8].

Under physiological conditions, the PM from capacitated sperm remains intact for an extended period and will not undergo a spontaneous fusion with the underlying outer acrosome membrane (OAM) unless an additional AE inducer is present [8]. Commonly, lysophosphatidylcholine, progesterone or calcium ionophores are used to induce AE *in vitro* [9]–[11]. Before the final execution of AE, SNARE (Soluble N-ethylmaleimide-sensitive factor Attachment protein Receptor) proteins from the acrosomal vesicle and the PM form a trimeric *trans*-SNARE complex that serves to position and execute the fusion of the two interacting membranes [8], [12], [13]. To this end, during AE, elevated intracellular calcium levels indirectly (via the interactions between calcium-bound synaptotagmin, complexin and the SNARE complex) cause a conformational change of the *trans*-SNARE complex into a *cis*-SNARE complex and thus the merging of the two docked membranes [14], [15]. In our lab, we have established that SNARE proteins migrate into the area where membrane rafts accumulate during sperm capacitation [16], [17]. Findings by Ackermann *et al.* support this as they reported that a multi-PDZ domain (a common structural protein subdomain found in many types of signaling proteins in a variety of cell types) protein MUPP1 is crucial for the relocalization of required proteins into detergent resistant membranes (DRM) and that this relates to the regulation of AE [18]. Hence, MUPP1 could play a role in recruiting SNARE and other essential proteins into the area where we have detected membrane raft clustering.

During *in vitro* capacitation of porcine sperm, the PM and the OAM are stably docked [8]. Despite this tight interaction, the two interacting membranes do not fuse in the absence of AE inducers and could even be isolated as bilamellar membrane structures after ell subfractionation [8]. The lack of spontaneous fusion of the acrosome with the PM strongly suggests that the trimeric *trans*-SNARE complex is stabilized with additional interacting protein(s) hindering the fusion machinery. Complexin, one of the cytosolic proteins which can interact with SNARE proteins or SNARE protein complexes is known to play dual functions in preventing and facilitating membrane fusion [19]–[22]. Recent findings indicate that complexin is able to halt membrane fusion by extending the accessory helix away from its central helix-bound SNAREpin and occupying the vacant R-SNARE binding site of a second SNAREpin on the opposite membrane; this disables the interacting SNAREs from complete zippering [23]. In the presence of calcium, complexin undergoes a conformational switch in which its accessory helix becomes almost in a parallel orientation with the central helix-bound SNARE complex and thus allows the calcium-bound synaptotagmin to initiate membrane fusion [15], [24]. Apart from the better-established SNARE-complexin interactions in synaptic fusion, such essential knowledge is lacking for AE in sperm. Nevertheless, complexin has been shown to regulate AE in mammals through the interaction with the calcium sensor synaptotagmin [14], [25]. Interestingly, the above mentioned multi-PDZ domain protein MUPP1 has recently been shown to interact with CaMKII [26] and this interaction prevents spontaneous AE. On the other hand, its release by Ca^2+^ calmodulin from the PDZ scaffolding protein is required to facilitate AE by precisely adjusting an increase in Ca^2+^ to synchronized fusion pore formation [26]. Therefore, the intracellular calcium concentration regulated by the above mentioned proteins might be crucial for the SNARE complex-complexin-synaptotagmin interactions and thus directly or indirectly be involved in sperm AE.

In the present study, we investigated whether or not the trimeric *trans*-SNARE complexes, which we have previously found aggregated in membrane rafts in capacitated sperm [8], [16], are stabilized by complexin and/or other interacting proteins. Moreover, the SNARE complex and interacting proteins found in mixed vesicles that are formed after calcium ionophore induced AE were identified. The role of sperm membrane rafts in the (de)stabilization of SNARE complexes and the relevant SNARE associating proteins that were found by proteomic detection are discussed with respect to their putative functions in sperm-zona binding and the membrane fusion events involved in AE.

## Results

### Acrosomal exocytosis and the formation of mixed vesicles is calcium dependent and facilitated by bicarbonate

**Figure 1:**
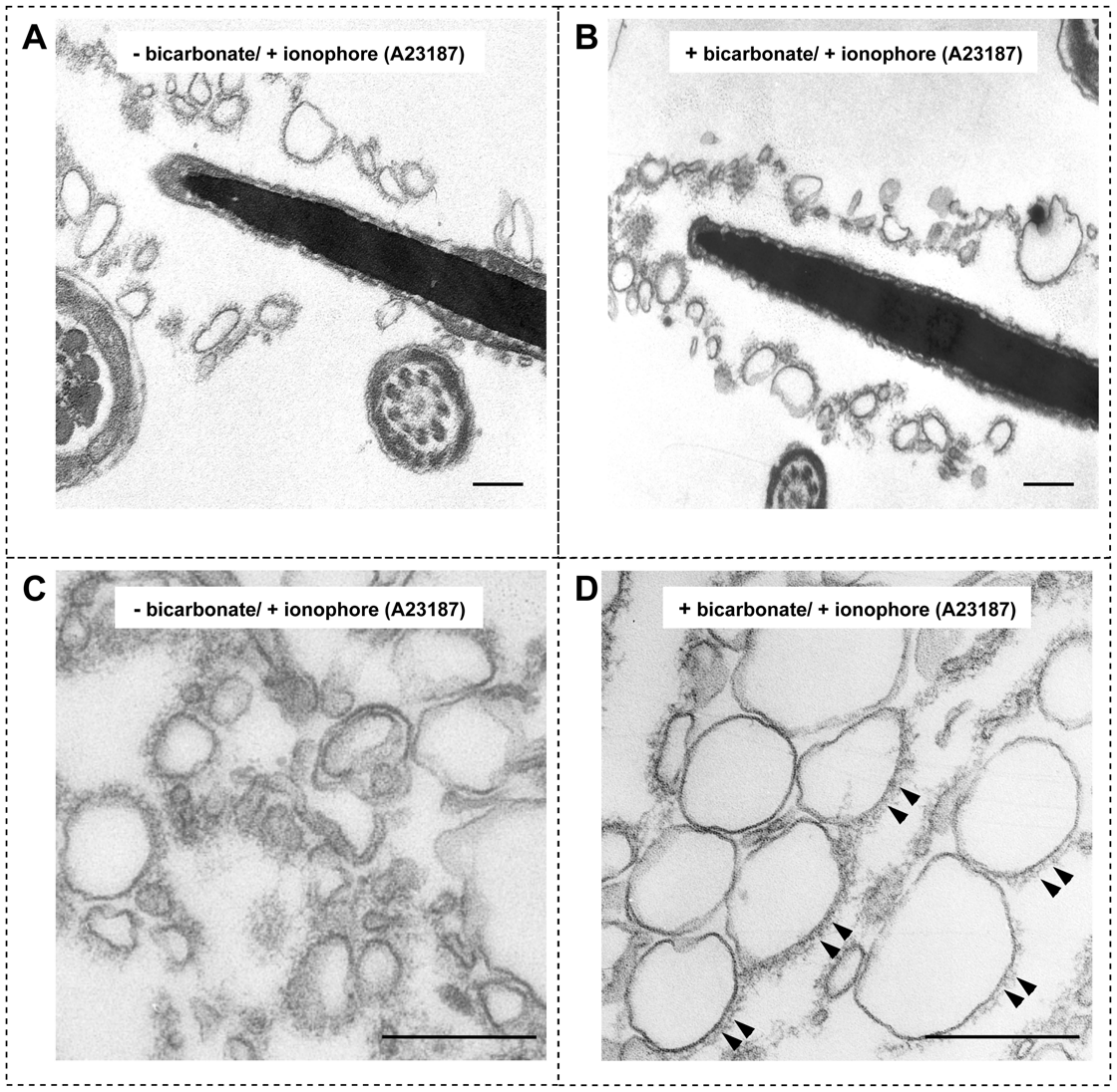
Ultrastructural morphology of acrosome reacted spermatozoa and the subsequently formed MVs. (**A**) MVs were present around the apical sperm head after AE was induced by calcium ionophore in the absence of bicarbonate. (**B**) The same was observed for sperm that were first capacitated in presence of bicarbonate and then challenged with Ca^2+^ ionophore. (**C**) MVs isolated from sperm treated as in panel A appeared as unilamellar membrane vesicle structures with a relatively large variation in size (126.7±54.5 nm) and in shape. (**D**) Unilamellar membrane vesicles isolated from sperm treated as in panel B showed a more homogeneous shape and size (184.4±28.5 nM). These MVs also appear to have more acrosomal matrix components associated to one side of the vesicles (arrow heads) as was already manifest before the MV shedding of the sperm head (see panel B). Statistical analyses on the morphology of MVs present in (C) and (D) were carried out by evaluating three sections from three independent samples (n = 3 values are indicated ± SD). Bar represents 200 nm.

AE was induced in incubated sperm cells treated with 5 µM Ca^2+^ ionophore A23187 in both control non-activated (Fig. 1A) and bicarbonate capacitated sperm (Fig. 1B), albeit that the efficiency of AE in capacitated sperm was much higher [16]. The resulting formation of unilamellar vesicles contained both PM and OAM material (see Tables 1, 2) and hence are called mixed vesicles (MVs). These MVs could be isolated from other sperm structures via differential centrifugation, and their ultrastructural appearance was visualized with transmission electron microscopy (TEM). MVs obtained under both conditions had a unilamellar membrane structure. However, in the absence of bicarbonate, MVs were less homogeneous in size (126.7±54.5 nm) and in shape (Fig. 1C) compared with the MVs formed in the presence of bicarbonate (average size 184.4±28.5 nm, Fig. 1D). Besides the differences in their ultrastructure, it appeared that in ionophore treated cells in the presence of bicarbonate, acrosomal material was present at only one side of the MVs. This was in contrast to the ionophore treated sperm in the absence of bicarbonate that resulted in a more homogeneous distribution of this material around the entire MVs surface (compare Fig. 1D with 1C; additional material layer is indicated with arrow heads).

**Table 1:** LC-MALDI MS-based identification of proteins from MVs derived from acrosome reacted porcine sperm.

Protein Name	Peptide Count	Accession Number	Sequence 1	Expect Value 1	Sequence 2	Expect Value 2	Database
Epididymal sperm-binding protein 1	9	ESPB1_PIG	AVYDGQWK	3,4E-05	YCLIEDYPR	5,0E-05	Swiss Prot v.57.10
Zona pellucida-binding protein 1*	5	ZPBP1_PIG	FFNQQVEVLGR	5,4E-07	IVGSPNFPVK	6,5E-05	Swiss Prot v.57.10
Carbohydrate-binding protein AWN	5	AWN_PIG	IFNSDGPQK	8,1E-06	QTIIATEK	3,4E-05	Swiss Prot v.57.10
Carbohydrate-binding protein AQN-3	4	AQN3_PIG	GSDDCGGFLK	2,1E-07	NYSGWISYYK	2,6E-07	Swiss Prot v.57.10
Acrosomal protein SP-10	2	ASPX_HUMAN	NQSFCNKI	2,6E-03	KIFEGGK	4,3E-03	Swiss Prot v.57.10
Sperm adhesion molecule 1 (SPAM 1)	2	Q8MI02_PIG	ESTALFPSIYLN	2,2E-04	QSIELVQQK	2,4E-02	Trembl v.40.10
Sperm inner acrosomal membrane protein IAM38*	2	Q2PMM0_BOVIN	FFNQQVEVLGR	1,2E-06	VYVMLHQK	5,1E-03	Swiss Prot v.57.10

Presented is a subset of the proteins identified for capacitated and acrosome reacted cells, that corresponds to sperm proteins previously reported in the literature to be relevant for sperm-oocyte interactions. Asterisks indicate identified acrosomal membrane proteins, others are plasma membrane specific.

* indicates acrosomal specific membrane proteins.

**Table 2:** LC-MALDI MS-based identification of other interesting proteins proteins from MVs derived from acrosome reacted porcine sperm.

Protein Name	Peptide Count	Accession Number	Sequence 1	Expect Value 1	Sequence 2	Expect Value 2	Database
Fibronectin*	15	FINC_HUMAN	SSPVVIDASTAID	6,9E-08	ESKPLTAQQT	5,2E-08	Swiss Prot v.57.10
Cumulus cell-specific fibronectin 1*	13	B8Y9T0_BOVIN	SYTITGLQPGTD	4,7E-10	VPGTSASATLT	8,1E-09	Trembl v.40.10
Acrosin*	10	ACRO_PIG	YVSGLEINDIALIK	1,2E-10	APQTCWVTG	2,0E-09	Swiss Prot v.57.10
Acrosin-binding protein*	10	ACRBP_PIG	AWQYLEDETLG	8,5E-12	LEQCHSETNL	9,6E-09	Swiss Prot v.57.10
Alpha-1-acid glycoprotein*	5	A1AG_BOVIN	WFYIGSAFR	1,5E-05	EFLDVIK	1,7E-04	Swiss Prot v.57.10
Alpha-2-HS-glycoprotein*	5	FETUA_BOVIN	QQTQHAVEGD	9,4E-11	QDGQFSVLF	6,1E-08	Swiss Prot v.57.10
Sperm-associated acrosin inhibitor*	5	IACS_PIG	KEPDCDVYR	4,4E-05	SHLFFCTR	7,7E-04	Swiss Prot v.57.10
Testis cDNA clone: QtsA-14886, similar to human acrosin binding protein	5	Q4R933_MACFA	TMSQLSSTLSR	2,6E-05	IYYENILLGVPR	2,1E-08	Trembl v.40.10
Alpha-1B-glycoprotein*	4	A1BG_BOVIN	ALWTGALTPGR	6,1E-05	FPLGPVTSTT	1,1E-04	Swiss Prot v.57.10
Arylsulfatase A*	2	Q8WNR3_PIG	TLFFYPAYPDE	4,6E-04	GYLTGMAGK	8,8E-04	Trembl v.40.10
Pancreatic secretory granule membrane major glycoprotein*	2	Q29209_PIG	YCTDPTTAIVENK	3,3E-07	LESTPQCNLR	7,7E-04	Trembl v.40.10
Spermatid-specific heat shock protein 70	3	Q9R2A1_MOUSE	TTPSYVAFTDT	2,0E-06	NQVAMNPQNT	6,5E-10	Trembl v.40.10
Acrosomal vesicle protein 1*	2	Q32KR2_BOVIN	NQSFCNKI	1,1E-03	NQSFCNK	8,1E-03	Trembl v.40.10
**Synaptotagmin-4**	1	SYT4_HUMAN	AFVVNIKEAR	8,8E-02			Swiss Prot v.57.10

Other interesting proteins identified in the MVs derived from bicarbonate capacitated and acrosome reacted cells based on similar criteria to Table 1. These are proteins that have been previously reported to have roles in secondary sperm-zona binding functioning. Proteins that were not detected in the capacitated membrane cavitates (Table 3) and are exclusively present in the MVs based on these analyses are highlighted in grey. Most of these proteins have been previously reported to be acrosomal membrane specific [29]. Protein ID was considered to be conclusive when two or more peptides were identified (e<0.05) (all proteins except the last protein in Table 2).

Protein ID was considered conclusive when two or more peptides were identified (e<0.05, all proteins above the line).

* : indicate acrosomal specific membrane proteins.

Highlighted in grey: Proteins that are not detected in the capacitated membrane cavitates and are exclusively present in the mixed vesicles.

Highlighted in bold: SNARE interacting protein.

### Docking and fusion of interacting membranes require different sets of SNARE proteins

**Figure 2:**
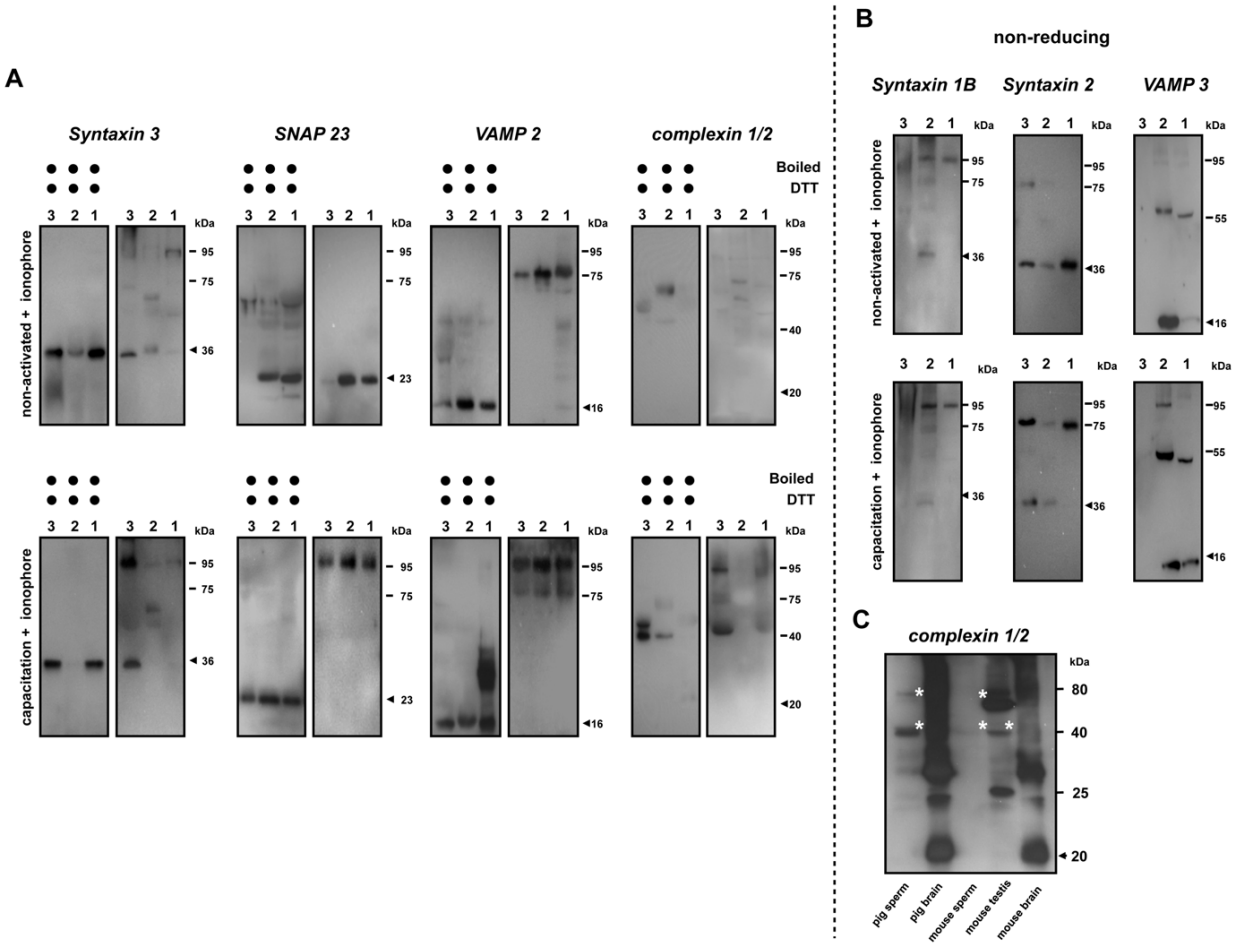
Bicarbonate and Ca^2+^ ionophore incubation results in MVs that contain high amounts of SNARE protein complexes. (**A**) When MVs were formed in the presence of bicarbonate and Ca^2+^ ionophore, a considerable amount of SNARE protein complex was present at 95 kDa (lower panels). A minimal amount of 95 kDa SNARE complexes was observed in MVs from the non-activated sperm when AE was induced by Ca^2+^ ionophore in the absence of bicarbonate (upper panels), although a 75 kDa VAMP 2 was found under this bicarbonate depleted condition. Besides the three interacting SNARE proteins found in the MVs, we also identified the capacitation-dependent participation of soluble complexin with the SNARE containing complex. (**B**) This shows the absence of previously reported SNARE proteins (to some extent syntaxin 1B is present in a 95 kDa complex but syntaxin 2 and VAMP 3 are not participating) in the non-activated MVs indicating that pre-docking and fusion of the acrosome with the PM requires different sets of SNARE proteins. (**C**) Identification of sperm and testis specific forms of complexin. To demonstrate that the unexpected 40 kDa complexin was not an artifact, whole sperm lysates and a mouse testis tissue homogenate were used. For the comparisons between different tissues, both pig and mouse brain homogenate were included. Despite the presence of multiple complexin positive bands, we found in both mouse and pig brain, a huge amount of complexin monomer was present. In contrast to the specific detection of 40 and 79 kDa complexin (marked with asterisks) in the sperm and testis, no 40 kDa complexin can be detected in the brain tissue of both species suggesting this unexpected 40 kDa complexin is a sperm-specific form rather than the artifact. A total of 35 µg crude protein was loaded to each lane of these Western blots from: 1: whole sperm cell lysate; 2: the remaining sperm head fraction (in the absence of MVs); 3: MVs. Samples were either incubated at the RT in the absence of reducing agent (non-reducing conditions) or boiled at 100°C for 10 min in the presence of 0.1 M DTT (reducing conditions). The arrows indicate the expected molecular weight of protein on the Western blot.

To investigate the participation of SNARE proteins during sperm AE, we characterized the migrating behavior of SNARE proteins on SDS-PAGE gels using (1) the parent whole sperm homogenate, (2) the remaining head fraction (in the absence of MVs) and (3) the MVs from both sperm samples that were incubated in absence and presence of bicarbonate and subsequently treated with calcium ionophore. In absence of bicarbonate, the monomeric forms of the plasma membrane specific SNARE protein syntaxin 3, and acrosome specific SNARE protein SNAP 23 were observed (Fig. 2A). A weak signal for a syntaxin 3 containing protein complex was also observed in the whole sperm homogenate, likely due to the variation between individual sperm in response to the treatment. In line with our previous observation, VAMP 2 already appeared in a protein complex at 75 kDa irrespective the capacitation process [8]. In contrast, MVs isolated from ionophore treated cells in presence of bicarbonate showed the pronounced presence of a SNARE containing protein complex at 95 kDa suggesting that an additional protein was associated with the 75 kDa trimeric *trans*-SNARE complex (Fig. 2A).

This 95 kDa SNARE containing complex was not observed in isolated membrane cavitates (membrane materials containing both PM and OAM from the apical sperm head) before the calcium ionophore treatment [8]. Intriguingly, syntaxin 1B and VAMP 3, two of the SNARE proteins that were responsible for the stable docking of acrosome to the PM upon sperm capacitation [8] were not recovered in the MV fraction (Fig. 2B). It is likely that the high Ca^2+^ levels during AE caused a dissociation of the docking SNARE complex and this suggested that the capacitation-induced stable docking of the OAM with the PM and the Ca^2+^-dependent fusions of OAM and PM are mediated by a different trimeric SNARE complex.

Besides the SNARE proteins, we also observed the exclusive participation of complexin 1/2 in this high molecular weight (MW) protein complex in the MV fraction that was recovered only in the bicabonate capacitated and subsequently Ca^2+^ ionophore treated sperm samples. Our results suggest a bicarbonate-dependent interaction between complexin 1/2 and the SNARE complex (syntaxin 3/SNAP 23/VAMP 2) that executes AE. Interestingly, under reducing conditions, complexin 1/2 did not dissociate from the SNARE complex into its supposed monomeric form at 19–20 kDa; but instead, a 40 kDa complexin positive signal was observed (mostly in the MV fraction, Fig. 2A). This 40 kDa form appears to be testis/sperm cell specific as both mouse and pig brain homogenate contained mainly the monomeric form of complexin 1/2 at the expected 20 kDa; while sperm and testis from both mouse and boar showed mainly the complexin 1/2 positive signals at the higher MW than the expected 20 kDa (marked with asterisks, Fig. 2C).

### Redistribution of SNARE interacting proteins upon capacitation and acrosomal exocytosis

**Figure 3:**
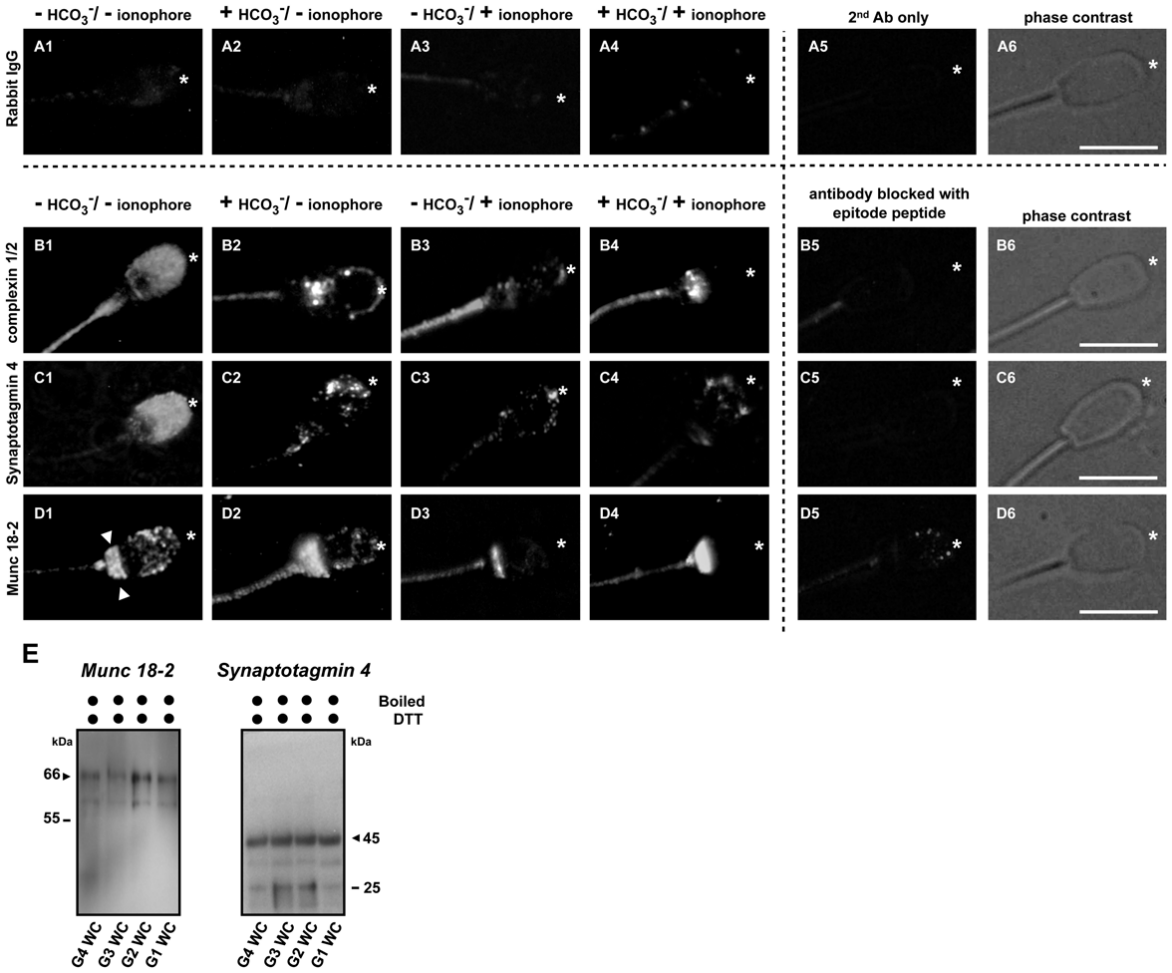
Bicarbonate-dependent relocalization of SNARE interacting proteins. (**A–D**) Immunofluorescence detection of SNARE interacting proteins complexin 1/2, synaptotagmin 4 and Munc 18-2 showed a homogenous distribution over the entire sperm head in the control, non-activated sperm (B1–D1). Munc 18-2 had an additional strong staining at the post-equatorial region (D1, indicated with arrow heads). Upon capacitation, these proteins relocalized to the apical sperm head (marked with asterisk) and appeared as punctate aggregates indicating their association with other proteins (B2–D2). When AE was induced (either in the absence [B3–D3] or in the presence [B4–D4] of bicarbonate), the majority of the signal at the apical ridge of the sperm head disappeared, which was due to the removal of apical membranes after shedding MVs from the sperm heads. A minor signal can still be detected in some sperm cells when AE is induced in the absence of bicarbonate while the majority of the signal was lost in the acrosome reacted sperm in the presence of bicarbonate; this is due to different efficiencies in AE induction. Purified rabbit IgG were used as a negative control (A1–A4) since these Abs were raised in rabbit. To demonstrate the specificity of the signals observed, sperm cells were also incubated in the absence of primary Ab (A5) or were incubated in the primary Ab that was immunized with specific blocking peptide of which the antibody was produced (B5–D5). Bars represent 10 µm. (**E**) Validation the presence of Munc18-2 and synaptotagmin 4 in porcine sperm. Whole sperm cell lysates (WC) from 4 different incubation conditions (G1–G4, see Methods and Materials) were used for the further validation on the presence of Munc 18-2 and synaptotagmin 4 observed in other independent experiments. The arrows indicate the expected molecular weight of protein on the Western blot.

The localization of SNARE interacting proteins in porcine sperm was assessed by indirect immunofluorescence microscopy. When sperm were incubated either with purified rabbit IgG (Fig. 3 A1–A4) or with secondary antibody alone (in the absence of primary antibodies, A5), we observed only a weak and non-specific background signal over the entire sperm cell. When sperm was incubated in the absence of bicarbonate (and without Ca^2+^ ionophore; protocol 4) both complexin 1/2 and synaptotagmin 4 showed a homogeneous diffuse staining pattern over the entire sperm head in the majority (>80%) of the sperm cells (Fig. 3 B1, C1), whilst Munc 18-2 had, besides the punctate staining over the entire sperm head, an additional strong staining at the post-equatorial region (indicated with arrow heads, Fig. 3 D1). When sperm was incubated in the presence of bicarbonate (and without Ca^2+^ ionophore; protocol 2), all SNARE interacting proteins redistributed towards the apical area of the sperm head (indicated with asterisks) and appeared in a punctuate staining pattern (Fig. 3 B2–D2) rather than the diffuse staining pattern observed in the absence of bicarbonate. Both complexin 1/2 and Munc 18-2 are cytosolic proteins and the observed aggregation of these proteins at the apical ridge of sperm head suggested a capacitation-dependent interaction of these proteins with SNARE proteins/complex. As expected, acrosome reacted sperm (after bicarbonate treatment and Ca^2+^ ionophore addition; protocol 1) had lost the majority of the immunofluorescence signals at the sperm head (Fig. 3 B4–D4) due to the shedding of MVs from the sperm head which is specific for the anterior sperm head area (Fig. 1). In line with this, in Ca^2+^ ionophore treated sperm in the absence of bicarbonate (protocol 3; i.e. where AE is much less efficient [16]), the immunofluorescence signals at the apical sperm head were still present in the majority (∼85%) of the non-capacitated but acrosome reacted sperm (Fig. 3 B3–D3). The specificities of the antibodies were tested by pre-incubating primary antibodies with synthetic blocking peptides which essentially diminished the immunosignals completely (Fig. 3 B5–D5). Western-blotting on these SNARE interacting proteins was also carried out to further validate the observed signals from the immunofluorescence studies (Fig. 3E).

### Capacitation-dependent complexin 2 interactions with the raft specific trimeric SNARE complex

**Figure 4:**
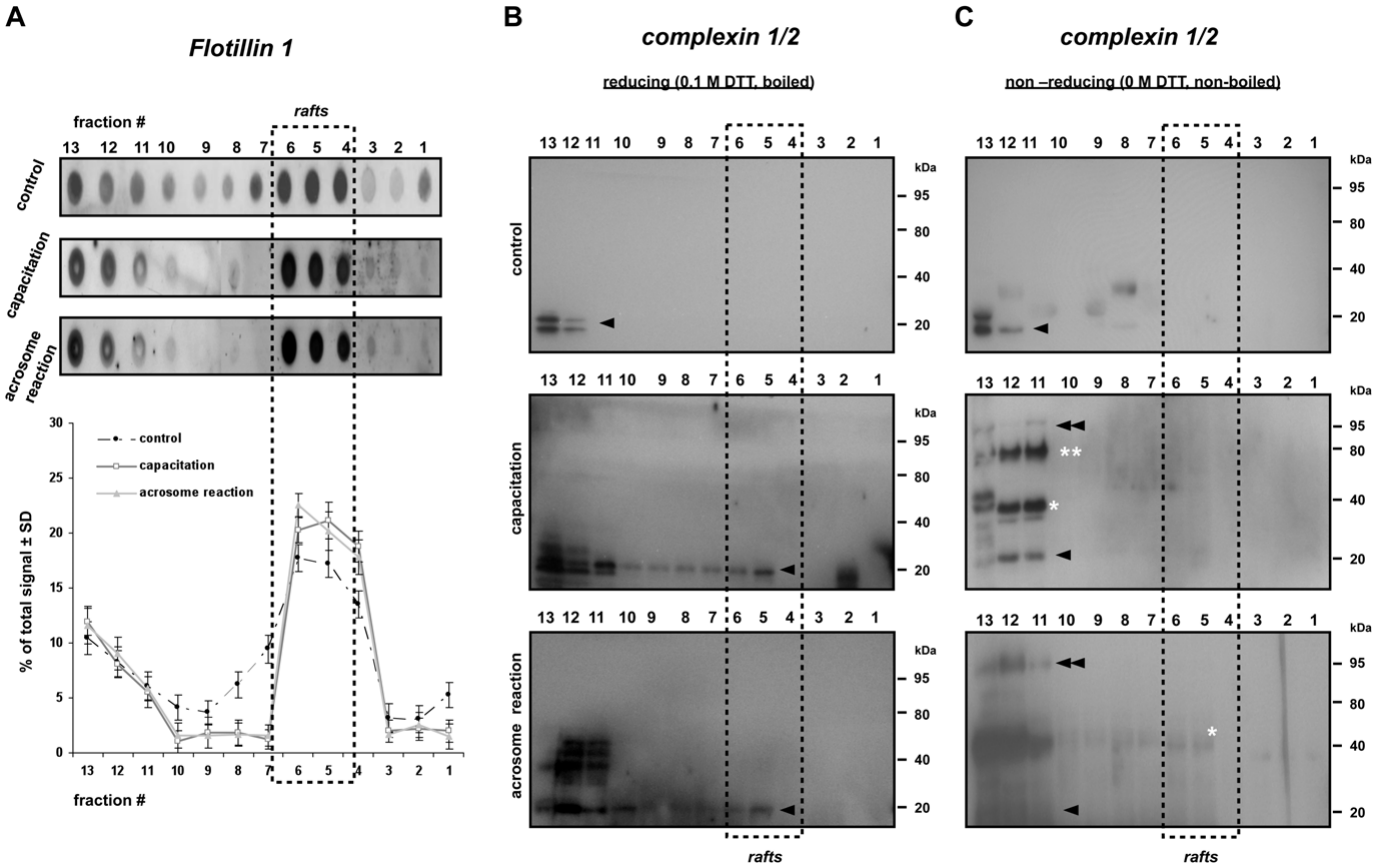
Capacitation induces complexin 2 interaction with the SNARE complex in both the raft and non-raft fractions. (**A**) Dot blots and density graphs for flotillin 1 in detergent treated sperm samples after separation over a discontinuous sucrose gradient. Detergent resistant membranes (DRM) were isolated using Triton X-100. DRMs were located at fraction 4–6 based on the enrichment of the raft marker protein flotillin 1. (**B**) Western blots of the gradient fractions 1–13 for complexin 1/2 under reducing conditions. Two monomeric forms (indicated with the arrow head) of complexin were detected at 19 (complexin 1) and 20 kDa (complexin 2) in porcine sperm. Both complexins were predominately present in the soluble fraction, no migration or complexed complexin can be detected in the control non-activated sperm (i.e. in the absence of bicarbonate; upper panel). When sperm cells were capacitated in the presence of bicarbonate, a clear migration of complexin 2 from the non-DRM fractions into the DRM fractions was observed suggesting the capacitation-dependent aggregation of complexin 2 to the apical area of the sperm head (middle panel) which remains in the DRM after the acrosome reaction (lower panel). (**C**) See (B) but these Western blots were performed on sample separated by SDS-PAGE under non-reducing conditions. Complexin 2 remains associated with different protein (complexes) than only with the trimeric SNARE complex. The 40 kDa complexin positive signal (indicated with an asterisk) indicated a specific dimer form of complexin in the porcine sperm. Further identification of 79 kDa complexin (marked with double asterisks) may reflect to the complexin-SNAREpin complex that does not have the full fusion ability and may mirror the complexin sub-population observed at the post-equatorial region of the sperm head in Figure 3. A major shift of the signal from 79 kDa to 95 kDa upon AE indicated the participation of the R-SNARE protein in these complexes.

To elucidate whether membrane rafts play a role in the SNARE proteins- complexin interaction, the widely applied detergent-based (Triton X-100) isolation protocol to isolate membrane rafts was used [27]. We first characterized membrane raft fractions from these Triton-treated sperm samples with the raft marker protein flotillin 1. Under all incubations, the floating DRM fractions #4–6 representing the membrane rafts carried approximately 48.5%, 60.2%, 60.5% of total cellular flotillin 1 (Fig. 4A).

Monomeric 19–20 kDa complexin 1 and 2 were detected (indicated with a single arrow head) in the detergent soluble fractions at the highest density side of the sucrose gradient (fraction #12 and #13) when sperm were incubated in the absence of bicarbonate and without Ca^2+^ ionophore treatment. When sperm was incubated with bicarbonate (irrespective of a subsequent Ca^2+^ ionophore treatment), a subfraction of complexin 2 was present in the DRM fractions (#4–6) (Fig. 4B). Under non-reducing conditions, when sperm were incubated in the presence of bicarbonate (irrespective of subsequent treatment with Ca^2+^ ionophore), we further detected additional complexin 2 containing bands likely reflecting self aggregation of complexin and/or association of complexin with its interacting proteins (at 40, 79 and 95 kDa, indicated with single asterisk, double asterisks and double arrow heads, respectively). These higher MW complexin containing bands were detected in the highest sucrose density fractions (#11–13; Fig. 4C). Our data suggested that, in absence of bicarbonate, complexin was predominantly present in its soluble and monomeric form. However, in the presence of bicarbonate, complexin 2 became associated with membrane proteins.

**Figure 5:**
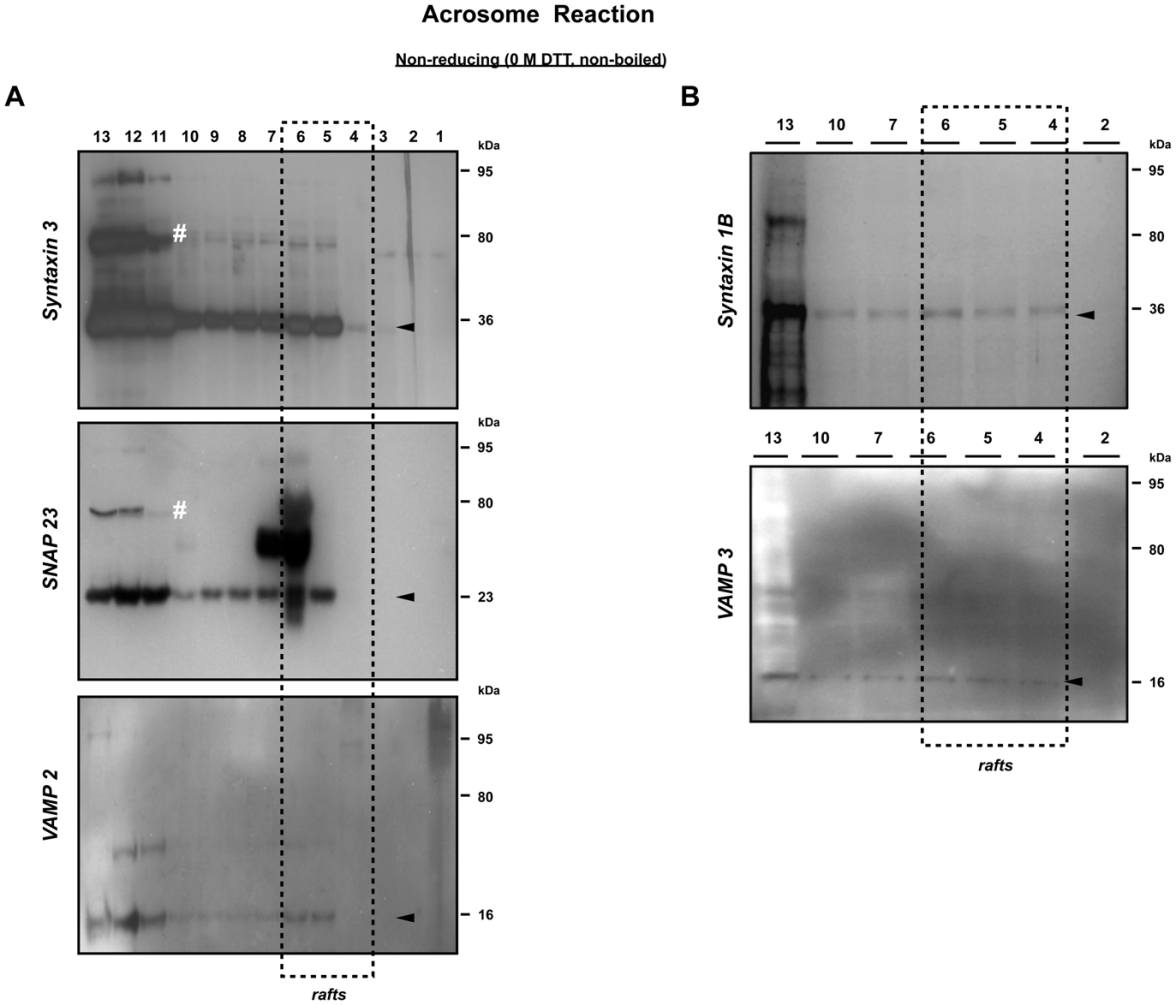
Identification of the complexin-SNAREpin complex in the non-raft fractions. (**A**) A Q-SNARE complex containing syntaxin 3 and SNAP 23 was detected on Western blots of the high sucrose gradient fractions (#11–13, indicated with number signs). This complex was found to lack the complementary R-SNARE VAMP 2 and thus enabled us to distinguish two different SNARE containing complexes in the porcine sperm. (**B**) The previously identified SNARE complex responsible for acrosome to plasma membrane docking (containing syntaxin 1B/SNAP 23/VAMP 3 [8]) was not observed in the same fractionated DRM sample at the high M.W. position (at 75 kDa) indicating the dissociation of this SNARE complex upon AE. Arrowheads indicate monomeric form of SNARE proteins present in these fractions. The arrows indicate the expected molecular weight of protein on the Western blot.

The 40 kDa complexin positive band reflects the germ-line specific stable dimeric form of complexin (Fig. 2C). We speculate that the 79 kDa complexin positive complex represents the recently reported pre-fusion SNAREpin-complexin complex that consists of two Q-SNARE proteins (syntaxin, SNAP) and complexin [22]. To investigate this possibility, we performed Western-blotting of the sucrose gradient fractions from sperm samples with antibodies against potential SNAREpin forming candidates. We indeed observed the same 79 kDa syntaxin 3 and SNAP 23 positive protein complexes in the non-DRM fractions (Fig. 5A, marked with number signs). More importantly, this 79 kDa protein complex had no affinity for anti-VAMP 2 antibodies. With these data we were able to discriminate between the SNAREpin-complexin complex (syntaxin 3/SNAP 23/complexin 2) and the trimeric form of SNARE complex (syntaxin3/SNAP 23/VAMP2) and showed the bicarbonate-dependent formation of both protein complexes in porcine sperm. In line with our earlier observation on the isolated MVs (Fig. 2), in Ca^2+^ ionophore treated (AE) sperm, we found a major bicarbonate dependent shift of the protein complex from 79 kDa to 95 kDa (Fig. 4C, Fig. 5A). The presence of this 95 kDa complexin-containing complex not only supported the findings of Figure 2, but also demonstrated that complexin was indeed interacting with the trimeric syntaxin 3/SNAP 23/VAMP 2 (to form the 95 kDa complex) and was equally efficient in binding syntaxin 3/SNAP 23 complex (to form a 79 kDa complexin-SNAREpin) prior to attracting VAMP 2 for the 95 kDa complex when Ca^2+^ ionophore was added. All together this data indicated the additional participation of the complementary VAMP 2 with the SNAREpin-complexin complex upon the rise of intracellular calcium levels that initiate AE. Interestingly, we were not able to recover previously identified SNARE protein complexes involved in acrosome to plasma membrane docking [8] in the same fractionated DRM samples, where only the monomeric form of syntaxin 1B and VAMP 3 were detected (Fig. 5B). The presence of these monomeric SNARE proteins (syntaxin 1B, VAMP 3) in the raft fraction and the absence of high M.W. docking SNARE complex suggests the dissociation of this “docking” SNARE complex during the formation of the SNARE complex that drives AE fusions and contains syntaxin3/SNAP 23/VAMP2.

### Calcium dependent dissociation of complexin 2 from the SNARE complex

**Figure 6:**
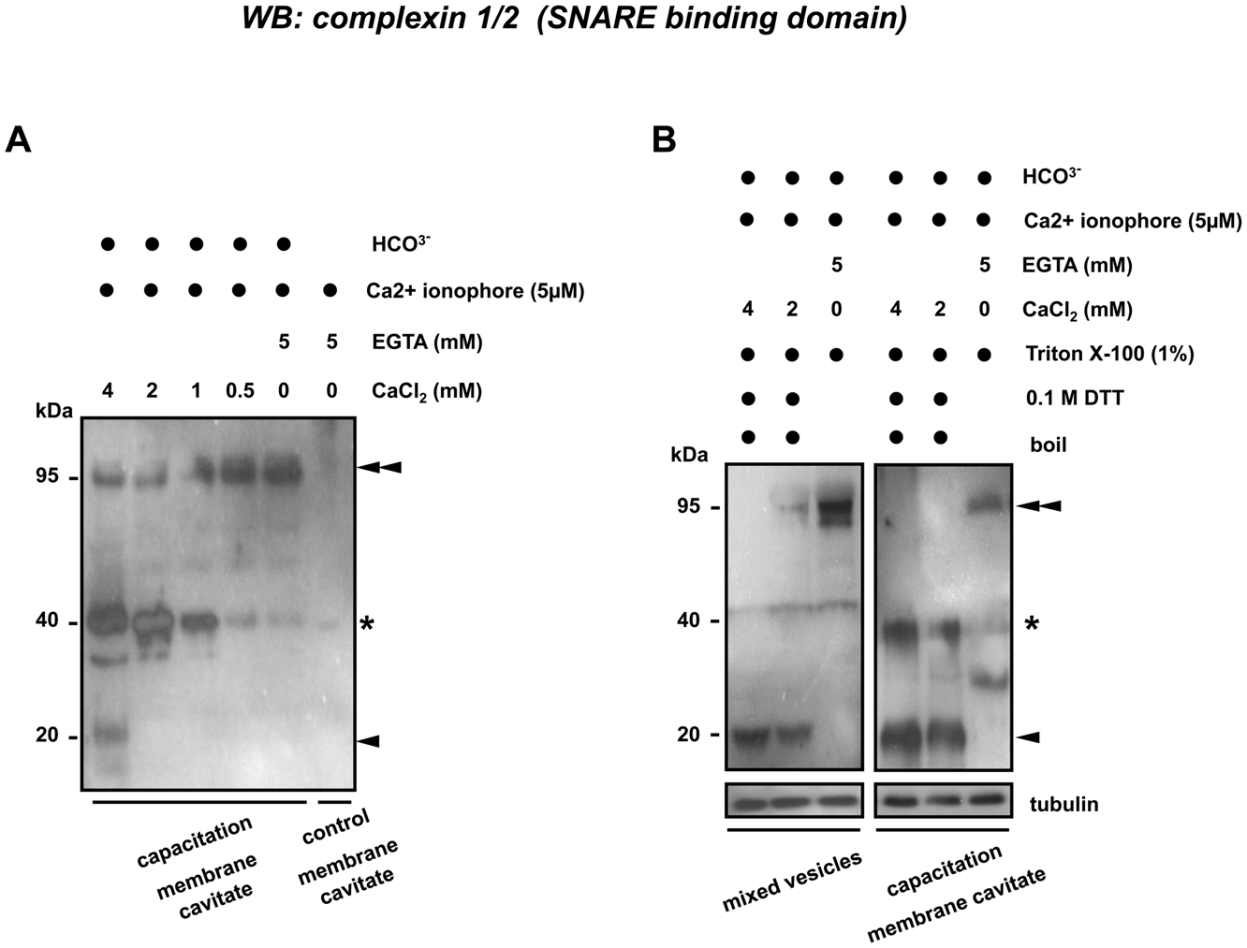
Calcium and bicarbonate dependent interactions of complexin to protein (complexes). (**A**) No complexin was recovered on Western blots from membrane cavitates obtained from the non-activated sperm. Complexin emerged at 95 kDa (double arrow heads) in the capacitated membrane cavitates. Complexin showed a calcium-dependent release into a 40 kDa dimer (marked with asterisk) and 20 kDa monomer (marked with single arrow head) as Ca^2+^ levels increased. (**B**) The unexpected stability of the 40 kDa complexin dimer was common to both membrane cavitates and MV samples and showed further dissociation into the expected monomer (arrow head) in the presence of >2 mM Ca^2+^ and 1% (v/v) Triton X-100.

Complexin is known to interact with synaptotagmin with changing cellular Ca^2+^ concentration [14], [15]. Therefore, we anticipated that the elevation of Ca^2+^ levels could lead to a different interaction between complexin and SNARE complexes. We incubated the isolated membrane cavitates (either “control membrane cavitates” containing apical plasma membrane [APM] from the non-activate sperms or the “capacitation membrane cavitates” containing both PM and OAM materials from the bicarbonate capacitated sperms [8]) with 0–4 mM CaCl_2_ in the presence of 5 µM Ca^2+^ ionophore. The absence of complexin in the control membrane cavitates showed the lack of complexed complexin at the apical area of the sperm head, supporting our data that the formation of a complexin containing SNARE protein complex is bicarbonate dependent. Interestingly, we found under non-reducing conditions that the majority of the complexin showed a Ca^2+^-dependent and concentration-dependent dissociation from the 95 kDa SNARE containing protein complex (Fig. 6A, indicated with double arrow heads). This indicated that a threshold intracellular Ca^2+^ concentration is required for the dissociation of complexin from the SNARE complex. However, the released complexin appeared first in a 40 kDa dimeric form (Fig. 6A, indicated with asterisk) as we found earlier in the MV samples (Fig. 2) and in the detergent soluble membrane fractions under non-reducing condition (Fig. 4C). In a sharp contrast to these findings, complexin in the DRM fractions under reducing conditions were exclusively recovered as monomers (Fig. 4B); therefore, we suspected that the addition of detergent (Triton X-100) we used during raft isolation lowered the stability of this 40 kDa complexin dimer. To test this, we incubated both APM and MV samples with different concentrations of CaCl_2_ in the presence of 1% Triton X-100. Indeed we found under these stringent reducing conditions, that the 95 kDa complexin containing protein complex (and the formed 40 kDa complexin dimer) fell apart into the expected monomer at 20 kDa (Fig. 6B, marked with single arrow head). We believe that this testis/sperm specific complexin isoform represents a tissue specific stabilized dimer of complexin. This dissociation was more efficient in the MV than in the membrane caviates suggests a tighter interaction of complexin with trimeric *trans*-SNARE complex in capacitated sperm.

### Proteomic identification of proteins in membrane vesicles

Using a proteomics approach we have determined the protein composition of membrane cavitates from bicarbonate capacitated sperm and identified VAMP 3, syntaxin binding protein 2 (also known as Munc18-2 or Munc18-b) and Rab 2A (Table 3, highlighted in bold). Note that not all proteins identified are included but rather only proteins that have previously been characterized in sperm cells or novel proteins with a potential role on SNARE mediated membrane fusion. The criteria to accept an identification for a protein were high probability scores (e<0.05) for ≥2 peptides found in that protein [28]. A lower confidence but still likely identification with a single peptide match e<0.05) was found for syntaxin 12. The membrane cavitates were highly purified (they contained >10 fold more specific PM material over the whole sperm lysate [8]) and a number of proteins routinely found to be specific for PM origin [29] were also detected in these vesicles (Table 3). Of particular interest was the detection of a part of the PDZ domain-containing protein (C16orf65, Table 3). Thus this protein could play a role in recruiting proteins (e.g. SNARE proteins or complexin) into the DRM fractions during capacitation and might be responsible for the regulation of AE [18].

**Table 3:** LC-MALDI MS-based identification of proteins from membrane cavitates from capacitated porcine sperm.

Protein Name	Peptide Count	Accession Number	Sequence 1	Expect Value 1	Sequence 2	Expect Value 2	Database
Zonadhesin#	17	ZAN_PIG	FVELQTAFGLR	2,8E-07	QEGVSCLSK	8,1E-06	Swiss Prot v.57.10
Epididymal sperm-binding protein 1# *	9	ESPB1_PIG	NCIVEGSFFGK	3,2E-06	TNSLSPWCATR	1,6E-06	Swiss Prot v.57.10
Carbohydrate-binding protein AWN# *	6	AWN_PIG	SSSNIATIK	5,7E-07	QTIIATEK	3,0E-06	Swiss Prot v.57.10
Carbohydrate-binding protein AQN-3# *	5	AQN3_PIG	GSDDCGGFLK	3,9E-07	NYSGWISYYK	2,6E-06	Swiss Prot v.57.10
ADAM3b#	4	A5HJZ3_PIG	GLLCVSAQLR	1,8E-04	NFDTQYTYYK	1,3E-06	Trembl v.40.10
Fertilin beta#	4	Q866A8_PIG	CHPNDLR	9,4E-03	TDESGACGLTA	1,2E-09	Trembl v.40.10
PDZ domain-containing protein C16orf65	4	CP065_BOVIN	DINCDVMIHR	2,4E-06	APSPYWTMVK	4,0E-03	Swiss Prot v.57.10
Acrosomal protein SP-10# *	3	ASPX_HUMAN	NQSFCNKI	5,2E-04	KIFEGGK	2,6E-02	Swiss Prot v.57.10
ADAM3a#	3	A5A4F6_PIG	QCAELFGK	1,1E-03	SEVVPFK	1,6E-02	Trembl v.40.10
CD44	3	CD44_BOVIN	YAGVFHVEK	1,2E-04	TEAADLCK	1,5E-04	Swiss Prot v.57.10
Carbohydrate-binding protein AQN-1#	2	AQN1_PIG	ISTYEGPK	1,3E-04	EYVEVQDGLP	8,0E-03	Swiss Prot v.57.10
**Ras-related protein Rab-2A**	2	RAB2A_HUMAN	GAAGALLVYDI	1,5E-04	MITIDGK	2,2E-02	Swiss Prot v.57.10
Sperm adhesion molecule 1 (SPAM1)# *	2	Q8MI02_PIG	ESTALFPSIYLN	8,5E-04	TFMQETLK	6,6E-03	Trembl v.40.10
**Syntaxin-binding protein 2**	2	Q2NL10_BOVIN	DLSHILK	2,7E-03	ADTPSLGEGP	1,6E-03	Swiss Prot v.57.10
**VAMP 3**	2	VAMP3_HUMAN	ADALQAGASQF	4,3E-17	LQQTQNQVD	9,7E-03	Swiss Prot v.57.10
Syntaxin 12	1	STX12_HUMAN	ISQATAQIK	3,9E-02	-	-	Swiss Prot v.57.10

Presented are subsets of those proteins identified that correspond to previously identified sperm proteins relevant to sperm-oocyte interactions [29]. SNARE-related protein identifications are highlighted in bold. In addition one proteins with a putative function in the recruitment of SNARE proteins into membrane rafts [26] is shown in red. Number signs indicate proteins that have been previously found in membrane cavitates of porcine sperm [29]. Asterisks indicate proteins that were identified in the MV preparations (Tables 1 and 2). Protein ID was considered to be conclusive when two or more peptides were identified (e<0.05) (all proteins above the line).

Protein ID was considered conclusive when two or more peptides were identified (e<0.05, all proteins above the line).

Highlighted in bold: SNARE proteins or SNARE interacting proteins.

Highlighted in red: Protein with putative function in the recruitment of SNARE proteins into the membrane raft area.

# : indicate proteins which are routinely found in the apical plasma membrane cavitates of porcine sperm.

* : indicate proteins that are recovered as plasma membrane proteins in the mixed vesicles.

We speculated that the additional layer observed in Fig. 1D (indicated by arrow heads) contained secretory proteins that were originated from the acrosome or acrosomal membranes. To investigate whether the capacitation process recruits specific acrosomal proteins to the MVs upon AE, we subjected isolated MVs to proteomic analysis. We detected not only major PM specific proteins but also membrane proteins unique to the acrosomal membranes (Table 1). This result was expected as the two interacting membranes merge their membrane proteins into the formed MVs. A number of proteins with glycoconjugate binding specificities were also recovered from the MVs isolated from bicarbonate-capacitated sperm and were not observed in MVs from non-activated sperm (Table 2). Most of them have been previously reported as acrosome specific proteins and some of them can be assumed to be of acrosomal origin as they were not recovered from the APM and are known to be secretory proteins. It is noteworthy that a number of the identified proteins have been previously reported to have a function in secondary sperm-zona binding (interaction and digestion of the zona pellucida (ZP) and are intra-acrosomal proteins from an acrosome reacted sperm), e.g. acrosin and its interacting proteins and arylsulfatase A [30], [31] (Table 2).

In the MVs, we identified synaptotagmin 4 albeit that this ID was not unequivocal based on our acceptance criteria listed above. Nevertheless, the presence of synaptotagmin 4 in porcine sperm was further confirmed with indirect immunofluorescence (Fig. 3C1–C4) and with Western-blotting (Fig. 3E). Synaptotagmin 4 is considered to be important for secretory membrane fusion as this protein belongs to a Ca^2+^ sensor protein family normally present on a secretory vesicle (the equivalent in sperm is the acrosome vesicle) that can interact with complexin and is known to be involved in the *trans* to *cis* configuration change of SNARE complexes and thus executes AE [14], [15].

## Discussion

In a previous study, we established the capacitation-dependent formation of a trimeric *trans*-SNARE complex (syntaxin 1B/SNAP 23/VAMP 3) that served to dock the OAM with the apical sperm PM [8]. Such docking resulted in the formation of a bilamellar membrane structure and this is a specific phenomenon for the lipid-ordered region of the sperm surface [16], [17]. This interaction did not result in spontaneous membrane fusions and no sign of AE was detected. In the present study, we extended our biochemical approach to elucidate the involvement and the interaction dynamics of SNARE proteins and complexin during sperm capacitation and AE.

Regulation of the onset of AE relies on the fine coordination between SNAREs and their interacting proteins as well as the precise adjustments of intracellular calcium levels [13], [26], [32], [33]. Here we have evoked AE by calcium ionophore treatment in non-activated and bicarbonate capacitated sperm. This resulted in the emergence of unilamellar vesicles which contained material from both membranes (PM and OAM) that fused at multiple sites. Despite the much higher incidence of AE after the bicarbonate incubation [16], we also found some AE in sperm that were Ca^2+^ ionophore challenged after incubation in absence of bicarbonate. These MVs differed morphologically from MVs isolated from bicarbonate treated cells after a Ca^2+^ ionophore challenge. The more homogeneous size and shape of MVs from bicarbonate-capacitated sperm when compared to control MVs may due to the specific involvement of membrane rafts during sperm capacitation induced AE. Their participation may lead to the formation of more well-organized micro-membrane structures [17], [18] and these raft structures could result in more uniform functional signal transduction platforms that are more efficient in facilitating the induction of AE. We also identified a number of membrane proteins with an acrosomal origin in the MVs derived from the bicarbonate and Ca^2+^ ionophore treated cells. It is possible that the previously reported capacitation-dependent SNARE complex formation at the lipid-ordered membrane microdomains [8], [16] recruits specific acrosomal membrane proteins to the area where acrosome fusion will later be executed. This capacitation-dependent recruitment may serve to leave the acrosome reacted sperm with secondary zona binding and digesting proteins fully exposed at the forefront of the zona interacting side enabling the immediate exposure of such proteins once the acrosome fusion is initiated by the zona pellucida (ZP).

In contrast to our previous finding [8] of a trimeric *trans*-SNARE complex in the capacitated sperm; in the acrosome reacted sperm, a 95 kDa protein complex was identified suggesting the association of additional protein(s) with the trimeric *trans*-SNARE complex. This newly identified 95 kDa protein complex contained not only three interacting SNARE proteins (syntaxin 3/SNAP 23/VAMP 2) but also a SNARE complex interacting protein, complexin 2. Of specific interest is the participation of complexin 2, but not complexin 1 in both porcine and human sperm upon AE, which is in contrast to the mouse showing species-differences in this process [14], [25]. The non- detection of the previously identified SNARE proteins syntaxin 1B and VAMP 3 in the MVs indicates that the bicarbonate-dependent formation of the acrosome docking trimeric SNARE complex [8] may not directly participate in the actual AE. However, it may facilitate the Ca^2+^-dependent formation of the other SNARE protein complex by bringing the two interacting membranes tightly together to allow the assembly of different sets of SNARE proteins for the execution of AE [34], [35]. The non-detection of the previously found “docking SNARE complex” (syntaxin 1B/SNAP 23/VAMP 3) in the MVs is likely to be due to a Ca^2+^-dependent disassembly of this “docking complex” prior to the sperm AE [13]. This idea is supported by the presence of monomeric syntaxin 1B and VAMP 3 in the raft fractions and the absence of a high M.W. docking SNARE complex. This intriguing phenomenon coincides with the Ca^2+^-dependent assembly of the other “fusion SNARE complex” (syntaxin 3/SNAP 23/VAMP 2) which remains intact in the MVs after the sperm AE. Therefore, future studies on the regulation and transition between docking and fusion-driving SNARE complexes will provide valuable insights in the regulation of the SNARE mediated fusion macheinery involved in the sperm AE.

SNARE protein-complexin interactions form the core and highly conserved machinery that drives intracellular membrane fusion in many different cell types [13], [19], [36]. Here we report for the first time that a 95 kDa protein complex from acrosome reacted porcine sperm contained not only three interacting SNARE proteins but also their interacting protein, complexin 2 while complexin 1 was present in sperm, but did not show this activity. In the absence of bicarbonate (non-activated sperm), complexin was only recovered in its soluble monomeric form and was not associated to any protein complexes. When sperm underwent capacitation in the presence of bicarbonate, complexin 2 became associated with SNARE containing protein complexes and could be co-purified in the membrane cavitates from the capacitated sperm. Interestingly, we found complexin 2 associated with more SNARE containing complexes than only with the trimeric *trans*-SNARE complex. In the present study, we were not able to identify the composition of complexin interacting proteins in other appearing complexin positive protein bands. However, we did show that complexin 2 was able to interact with both the pre-fusion SNAREpin complex and the complete trimeric SNARE complex after bicarbonate stimulated capacitation and a Ca^2+^ ionophore challenge.

The detection of the 79 kDa complexin 2/syntaxin 3/SNAP 23 complex in the DRM samples from acrosome reacted sperm mirrored the recently reported SNAREpin-complexin complex (which contained a Q-SNARE complex and a single complexin [23]). The fusion ability of these pre-fusion SNAREpin complexes is temporarily halted due to the lack of an R-SNARE binding site (occupied by the accessory helix of complexin, [23]). More importantly, these 79 kDa protein complexes were found mostly in the non-raft fractions and may reflect the complexin sub-population observed at the distal surface of the sperm head (Fig. 3) in the capacitated sperm. These temporarily clamped fusion complexes could act to prevent the fusion of membranes at this region as this posterior surface of the sperm head is specifically required for the fertilization fusion of sperm and the oolemma [37].

Another interesting observation was the profound localization of Munc 18-2 at the same distal surface region of the sperm head. Munc 18 is known to bind the free monomeric syntaxin and form a “closed position” to prevent the formation of SNAREpin or complete SNARE complex for further fusion [38]. This unexpected but specific localization of Munc 18-2 could also serve to prevent the fusion at this region which physiologically makes sense as this area of the sperm head is directly covering the nuclear envelope and fusion would open the nuclear content to the extracellular environment. These sub-populations of complexin 2 and Munc 18-2 found in the posterior area of the sperm head could create a double secure locked system to prevent unwanted membrane fusion and ensure the restricted binding and fusion sites at the apical area of the sperm head before their encounter with the oocyte.

The interaction between complexin and a trimeric SNARE complex is thought to be calcium-dependent [15], [22], [36]. Therefore we believe that Ca^2+^ influx into the sperm cell (normally evoked after sperm-zona binding) results in the dissociation of complexin. We indeed demonstrated that complexin 2 showed a calcium- and calcium concentration-dependent dissociation from the 95 kDa protein complex. However, the dissociated complexins found in porcine sperm required more stringent reducing conditions and a higher calcium concentration for their full dissociation from a dimeric into a monomeric form. We did observe a pronounced redistribution of complexin 2 to the apical sperm head and into the raft-specific fractions in capacitated and acrosome reacted sperm cells. Therefore, this unusual stability may due to the additional capacitation-dependent protein-protein interactions or the raft-specific binding of complexin with the *trans*-SNARE complex. The weak detection of the raft specific complexin-containing complex might be explained by the alteration of the binding epitode or the reduced accessibility for the antibody during the DRM isolation where it may have become cryptic for the antibody under the artificial Triton-treated conditions.

From the proteomic analysis of the MVs, we found a number of proteins from the OAM and luminal matrix are only recovered in the MVs from sperm that were bicarbonate treated and subsequent challenged with Ca^2+^ ionophore. Some of the identified proteins (e.g. ZP binding protein 1, AQN-3) have affinity for glycosylated proteins (e.g. ZP proteins) and thus may serve to establish the firm secondary sperm-zona binding after AE [29]. The fact that such proteins were not detected in MVs from control sperm (bicarbonate depleted sperm that were Ca^2+^ ionophore challenged) provides an indication that the sperm surface rearrangement and the concomitant reordering of the interacting OAM are functionally relevant for secondary sperm-zona binding. The proteomic data also identified a number of proteins that are known to be involved in the regulation of SNARE-mediated exocytosis (e.g. Rab 2A, syntaxin binding protein 2 [Munc 18-2], synaptotagmin 4 [a Ca^2+^ sensor known to be important for the execution of secretory vesicle fusion]).

Indeed, apart from the proteomic detection of above mentioned SNARE regulators, we observed the similar capacitation-dependent redistribution of these regulating proteins to the same apical area of the sperm head where SNARE proteins (syntaxin 1A/B, syntaxin 2, VAMP) [16] and the raft marker protein flotillin 1 [17] have been detected during sperm capacitation. Moreover, SNARE regulators were no longer detected at the apical ridge in the acrosome reacted sperm which strongly suggests their incorporation with the SNARE protein complex and their release from the sperm surface during the shedding of the MVs in which they are then captured after AE.

In summary, we have detected a specific set of SNARE proteins (syntaxin 3/SNAP 23/VAMP 2) that form a trimeric SNARE complex upon the induction of AE. This newly identified SNARE complex differs from the previously identified syntaxin 1B/SNAP 23/VAMP 3 complex that is responsible for the stable docking of acrosome to the PM. The binding and the dissociation of complexin 2 from this VAMP 2 containing SNARE complex demonstrates the dynamic interactions between different trimeric SNARE complexes and complexin 2 upon capacitation and AE. Moreover, apart from interacting with trimeric SNARE complexes, a separate complexin sub-population interacted with the pre-fusion SNAREpin and, by doing so, likely serves to prevent the preliminary fusion of the membranes at the non-apical sperm head area. When AE was induced by a Ca^2+^ ionophore, complexin 2 dissociates from the SNARE complex and allows the participation of complementary R-SNARE for the completion of AE. We postulate that the specific docking of the acrosome with the sperm surface is required to recruit certain secondary zona binding proteins at the surface as soon as AE is initiated by the ZP. We have identified a number of secondary zona binding proteins in MVs derived from bicarbonate capacitated sperm that in contrast were not observed in MVs from control non-activated sperm. The additional SNARE interacting proteins identified in the MVs of capacitated sperm indicate that capacitation and AE are more complicated and depend on more events than a simple interplay between the Ca^2+^ sensing protein, complexin and the SNARE complex. The recruitment of specific proteins and molecules during capacitation ensure the successful execution of AE, the penetration of ZP and subsequent sperm-oocyte binding.

## Materials and Methods

### Reagents and antibodies

All chemicals were obtained from Sigma (St. Louis, MO, USA) unless otherwise stated. All antibodies against SNARE proteins and SNARE associating proteins (complexin 1/2, Munc 18-2, synaptotagmin 4) were obtained from Synaptic Systems (Göttingen, Germany) unless otherwise stated. The anti-complexin 1/2 antibody used in this study is specifically against the SNARE complex binding domain where the immunogen is located inside the mapped binding domain of complexin 2 to the SNARE complex (aa 45–81). Mouse IgG1 monoclonal antibody against the membrane raft specific protein flotillin1 was obtained from BD Biosciences (San Jose, CA, USA). Ca^2+^ ionophore (A23187) was from Sigma.

### Sperm preparation

Freshly ejaculated sperm cells from highly fertile boars (*Sus scrofa domestica*) were obtained from a commercial breeder (Cooperative Center for Artificial Insemination in Pigs, ‘Utrecht en den Hollanden’, Bunnik, the Netherlands). Sperm cells were washed through a discontinuous (70% (v/v) and 35% (v/v)) Percoll gradient (GE Healthcare, Diegem, Belgium) as previously described [16]. All solutions were iso-osmotic (300±5 mOsm/kg) at room temperature (RT) and contained protease inhibitors (EDTA free, Roche, Mannheim, Germany).

1×10^9^ matured sperm cells per condition were used for the following *in vitro* fertilization (IVF) compatible protocols: (1) capacitation and AE: sperm cells were incubated in HEPES-buffered Tyrode's medium (HBT: 90 mM NaCl, 21.7 mM lactate, 20 mM HEPES, 5 mM glucose, 3.1 mM KCl, 1.0 mM pyruvate, 0.4 mM MgSO_4_, 0.3 mM NaH_2_PO_4_, 2 mM CaCl_2_, 100 µg/ml penicillin-streptomycin sulphate) with the addition 15 mM NaHCO_3_ in a open-capped tube at 38.5°C in a humidified atmosphere with 5% CO_2_ for 2.5 hours to induce capacitation. The AE of the capacitated sperm was subsequently induced by the addition of 5 µM calcium ionophore A23187; (2) capacitation: sperm cells were stimulated by incubation in HBT supplemented with 15 mM NaHCO_3_ in open vials for 2.5 hours at 38.5°C in a humidified atmosphere with 5% CO_2_; (3) non-activated but AE induced: sperm cells were incubated under the same protocol as condition 1, only without NaHCO_3_ in the capacitation incubation medium; (4) control non-activation incubation: sperm cells were incubated in HBT medium with the omission of bicarbonate in air-tight vials for 2.5 hours at 38.5°C in a tube rack place pre-warmed water bath. All incubation media were prepared in the presence of the cholesterol acceptor bovine serum albumin (0.3% (w/v) BSA in HBT: delipidated fraction V; Boehringer, Mannheim, Germany).

### Transmission Electron Microscopy (TEM)

Sperm cells as well as MVs from both treatments (protocol 1 and 3) were pelleted and fixed overnight at 4°C in Karnovsky (2% (v/v) paraformaldehyde with 2.5% (v/v) glutaraldehyde, diluted in cacodylate buffer) fixative. Pellets were washed with 0.1 M Na-cacodylate (pH 7.4) and post-fixed with 1% (v/v) osmium tetraoxide in 0.1 M Na-cacodylate (pH 7.4) for 1 hour. After washing with milli-Q H_2_O, pellets were incubated with 2% (w/v) uranylacetate for 1 hour. Fixed pellets were subsequently dehydrated in graded series of acetone (50%–100%) and embedded in Durcupan ACM resin (Fluka, Bachs, Switzerland). Ultrathin sections of 50 nm were obtained on a Reichert UltracutS (Leica Aktiengesellschaft, Vienna, Austria) and studied using TEM (Philips CM10, Philips, Eindhoven, the Netherlands).

### Membrane vesicle isolation

Apical membranes from capacitated (in presence of 15 mM bicarbonate; protocol 2) and non-activated (in absence of bicarbonate; protocol 4) sperm cells were obtained via nitrogen cavitation as previously described [39]. 1×10^9^ Percoll-washed sperm cells in TBSS buffer (Tris buffered sucrose solution; 5 mM Tris, 250 mM sucrose, pH 7.4) were subjected to a cell disruption device (Parr Instruments, Moline, IL, USA). The cavitate was slowly extruded and centrifuged at 1000*g* for 2 times 10 min at 4°C. Supernatants were combined and centrifuged at 4°C for 10 min at 6000*g*. The supernatant from the 6000*g* centrifugation was carefully loaded on top of a 80% (w/v) sucrose layer in SW 60 (Beckman, Palo Alto, CA, USA) tubes to prevent the stickiness of membrane materials at the bottom of the tube. Membrane materials were pelleted by ultracentrifugation at 4°C (2 times 70 min at 285000*g*). Membrane pellets were re-suspended in HBS (HEPES buffered saline; 5 mM HEPES, 2.7 mM KCl, 146 mM NaCl, pH 7.4). The remaining sperm heads (pellets that resulted from the 1000*g* centrifugation described above), sperm tails (pellets that resulted from the 6000*g* centrifugation described above) and the cavitated membrane fraction (after the 285000g centrifugation) were flash-frozen in liquid nitrogen and stored at −20°C for later use.

Mixed vesicles were isolated by differential centrifugation at 4°C as described above following sperm incubation under the required protocol. This was either incubation in the presence of 15 mM bicarbonate followed with a Ca^2+^ ionophore challenge (protocol 1) or incubation in absence of bicarbonate followed by Ca^2+^ ionophore challenge (protocol 3). Sperm cells from both conditions were subjected to a three-step centrifugation (1000*g*, 10 min; 6000*g*, 10 min and 285000*g*, 70 min) supernatants were carefully removed with the final 1 ml discarded. The remaining material was resuspended with HBS and subjected to a second ultracentrifugation (285000*g*, 70 min). Pellets from the second centrifugation (contains mixed membrane vesicles) were resuspended in ice-cold HBS, flash-frozen in liquid nitrogen and stored at −20°C. Purity of all membrane isolates were checked as previously described [40]–[42].

### Detergent Resistant Membrane (DRM) Isolation

To investigate the role of membrane microdomains (membrane rafts) in SNARE protein-complexin interaction dynamics, a detergent based extraction isolation protocol using ice-cold Trixon-X 100 was applied according to van Gestel et al. [17]. Sperm cells were washed in HBS, lysed in 2-(N-morpholino) ethanesulfonic acid (Mes) buffer (25 mM Mes, 150 mM NaCl, 1 mM EGTA), 1% (v/v) Triton-X 100; pH 6.5) and kept on ice for 30 min. The suspension was subsequently mixed with an equal volume of 80% (w/v) buffered sucrose solution. The mixture was overlaid with 8 ml 30% (w/v) sucrose followed by 4 ml 5% (w/v) sucrose in Mes buffer and centrifuged for 18 hour at 200000 g at 4°C. The DRMs appeared as an opalescent band in the low-density fraction of the gradient. Starting from the top of the gradient 1 ml fractions were collected for further analysis.

### Immuno-blotting

Protein concentrations were standardized according to the Coomassie Plus- Bradford™ Assay Kit (Pierce, Rockford, IL, USA). For membrane vesicle samples, an equal amount of total protein extract was resuspended with an appropriate amount of lithium dodecyl sulfate (LDS) loading buffer (Invitrogen, Carlsbad, CA) and incubated either at RT in the absence of reducing agent or heated for 10 min at 100°C in the presence of 0.1 M dithiothreitol (DTT) prior to immuno-blotting. For detergent resistant membrane experiments, an equal volume (15 µl) of different fractions was used. For the complexin-CaCl_2_ experiment, membrane cavitates were pre-incubated with 0–4 mM CaCl_2_ at 37°C for 20 min prior to the mixing with the loading buffer. Proteins were separated by SDS-PAGE (4% stacking and 10% running gel) and wet-blotted onto nitrocellulose membranes (Protran BA 85, Whatman, Dassel, Germany). After blocking for 1 hour with ReliaBLOT® Block (Bethyl Lab., Inc., Montgomery, TX, USA) at RT, blots were incubated with primary antibodies diluted in ReliaBLOT® overnight at 4°C. After washing the blots in TBS (5 mM Tris, 250 mM sucrose, pH 7.4) with 0.05% v/v Tween-20 (TBST), secondary antibodies were subsequently added for 1 hour. After rinsing with TBST, protein was visualized by using chemiluminescence (ECL-detection kit; Supersignal West Pico, Pierce, Rockford IL, USA).

### Dot-blotting

Dot blotting was performed by using the Easy-Titer™ ELIFA dot blot system (Pierce, Rockford IL, USA). Nitrocellulose membranes were rinsed in Milli-Q water and equal volumes of DRM fractions were subsequently pipetted into separate wells. Membranes and adhering proteins were dried using a vacuum system (flow rate 100 µl/1.5 min/well). Immuno-blotting procedures were followed as described above. Quantitative analysis of dot blot labeling was performed by scanning the blots with a GS-700 densitometer (Bio-Rad Laboratories, Hercules, CA, USA) using Quantity One acquisition software (version 4.3, Biorad). Densitometric quantitation was carried out with the use of Gel-Pro Analyzer software (version 3.0, MediaCybernetics, Silver Spring, MD, USA). Statistical analyses were performed with the use of SPSS 12.0 statistical software (SPSS Inc., Chicago, IL, USA).

### Indirect immunofluorescence staining

After the required incubation, sperm cells were subsequently fixed (4% (v/v) paraformaldehyde [Electron Microscopy Sciences, Hatfield, PA, USA]+0.2% (v/v) glutaraldehyde) at RT for at least 90 min. Fixed sperm cells were washed 2 times (600*g*, 5 min) in TBS and the fixative was further quenched with blocking buffer with glycine (TBST supplemented with 1% (w/v) BSA, 5% (v/v) normal goat serum and 100 mM glycine) for 15 min at RT. Permeabilized sperm cells (with 0.1% (v/v) Triton X-100, 30 min, RT) were washed with TBST and blocked in blocking buffer (TBST supplemented with 1% (w/v) BSA, 5% (v/v) normal goat serum) for 1 hour at RT. Sperm cells were subsequently incubated with primary antibodies (dilution in blocking buffer) overnight at 4°C on an end-to-end rotor. After extensive rinsing, cells were incubated with secondary antibody (donkey anti rabbit Alexa-488, 1∶100 dilution in blocking buffer) for 1 hour at RT in the dark. Finally, cells were rinsed in TBST, mounted in Vectashield (Vector Lab, Orton Southgate, Peterborough, UK) on Superfrost Plus microscope slides (Menzel, Braunschweig, Germany) and under a coverslip All samples were evaluated with an Olympus BH2 microscope. ImageJ (NIH; http://rsb.info.nih.gov/ij/) software was used for analysis of the images. Background subtraction and contrast/brightness enhancement (up to ∼20% enhancement using the maximum slider in Image J) were performed identically for all images in the same experiment.

To demonstrate the specificity of the fluorescence signals observed in this study, 1 µg/ml primary antibodies were blocked with 5 µg/ml synthetic peptide epitodes of the corresponding proteins the antibodies were raised against. The synthetic blocking peptides for anti-complexin1/2 antibody (for the C-terminus sequence: KYLPGPLQDMFKK), anti-Munc18-2 (sequence: DTLKKLNKTDEEISS), anti-synaptotagmin (sequence: DDDAETGLTDGE) and anti-flotillin1 (sequence: C-SISQVNHKPLRTA) were co-incubated with the corresponding antibodies for 2 hours prior to their use for immunofluorescence experiments. Negative controls were conducted by using either purified mouse or rabbit IgG. Fresh and Percoll-washed sperm were used in these series of experiments and at least two hundred sperm cells were examined and representative images were presented.

### LC-MALDI MS-based protein identifications of membrane vesicles

Two types of membrane vesicles were used for the proteomic analyses; (1) membrane cavitates from capacitated sperm were prepared as described above and previously [8], (2) MVs from capacitated and acrosome reacted sperm (protocol 1) were prepared as described above. Suspensions containing membrane vesicles were pelleted at 118,000*g* for 45 min at 4°C in a TLA-110 rotor, Optima-Max Ultracentrifuge (Beckman Coulter, Palo Alto, CA, USA). The pellets were solubilised in 100 µl triethylammonium bicarbonate (TEAB) lysis buffer (20 mM TEAB) containing 20 mM dithiothreitol (DTT) and 1% (w/v) SDS at RT for 10 min, and then heated to 95°C for 10 min. The samples were left at RT for 10 min before samples were subjected to protein precipitation using a 2D Clean-up kit (GE Healthcare, Buckinghamshire, UK). The pellets were resuspended in 20 mM TEAB and left overnight at 4°C. The protein content was then determined using a bicinchoninic acid (BCA) protein assay kit (Sigma, St. Louis, MO, USA). Samples were then reduced, denatured and alkylated using an Applied Biosystems iTRAQ (isobaric tag for relative and absolute quantitation) labelling kit with the standard protocol (Foster City, USA). The proteins were subjected to digestion with trypsin (ratio of 1 µg trypsin: 50 µg of sample), and incubated at 37°C for 12–16 h. The samples were then dried and resuspended in water with 0.1% (v/v) trifluoroacetic acid (TFA).

Digested peptides were separated on a nano-LC (liquid chromatography) system (UltiMate 3000, Dionex, Sunnyvale, USA) using a two-dimensional salt plug method as previously described [28]. Mass spectrometry was performed using an Applied Biosystems (Warrington, UK) 4800 MALDI (Matrix-assisted laser desorption/ionization) TOF/TOF (time-of-flight) mass spectrometer as previously described [28]. The tandem mass spectrometry (MS/MS) data was used to search the Swiss-Prot database (Version 57.10; mammalian taxonomy) in the first instance but also the Trembl database (Version 40.10; mammalian taxonomy) using the MASCOT database search engine v2.1.04 (Matrix Science Ltd, London, UK) embedded into GPS Explorer software v3.6 Build 327 (Applied Biosystems) (default GPS parameters, 1 missed cleavage allowed, fixed modification of MMTS(C), variable modifications of oxidation (M), pyro-glu (N-term E) and pyro-glu (N-term Q), 150 ppm mass tolerance in MS and 0.3 Da mass tolerance for MS/MS which are recommended published tolerances for LC-MALDI [28]. A minimum of two peptides with MASCOT e-values less than 0.05 was considered to be conclusive for protein identification.
